# Increased risk for thromboembolic events from combination of a gynecologic malignancy with severe acute respiratory syndrome coronavirus 2 infection: a case report

**DOI:** 10.1186/s13256-022-03340-8

**Published:** 2022-03-22

**Authors:** Alexandra Stefan, Marija Petkovic, Alexander König, Julian Koch, Friederike Hagemann, Rachel Wuerstlein, Nadia Harbeck, Sven Mahner, Till Kaltofen

**Affiliations:** 1 Department of Obstetrics and Gynecology, University Hospital, LMU Munich, Munich, Germany; 2 Department of Medicine IV, University Hospital, LMU Munich, Munich, Germany

**Keywords:** Ovarian cancer, Gynecologic malignancies, COVID-19, SARS-CoV-2, Thromboembolism, Review, Case report

## Abstract

**Purpose:**

During the severe acute respiratory syndrome coronavirus 2 pandemic, several patient groups are at particular risk. Mortality is higher among cancer patients and may be increased further by thromboembolic events, which are more common in coronavirus 2019 patients according to recent publications. We discuss the association of gynecologic malignancies, Severe acute respiratory syndrome coronavirus 2, and thromboembolism by reporting a case study and summarizing available literature.

**Case report:**

A 71-year-old Caucasian patient with ovarian cancer receiving first-line chemotherapy was diagnosed with deep vein thrombosis and pulmonary embolism. Routine screening revealed infection with severe acute respiratory syndrome coronavirus 2 in absence of specific symptoms. After uneventful recovery, oncologic treatment could be continued a few weeks later.

**Methods:**

We performed a systematic review of the literature on PubMed following Preferred Reporting Items for Systematic reviews and Meta-Analyses guidelines. The search included articles ahead of print, published between 1 December 2019 and 1 June 2020. Cross-searches were conducted on all relevant articles.

**Results:**

We identified five articles meeting the defined criteria, including two retrospective studies, a review, a position paper, as well as a letter to the editor.

**Conclusion:**

Cancer patients infected with severe acute respiratory syndrome coronavirus 2 have a relatively poor outcome, which may partially be due to a higher rate of thromboembolic events. Thromboprophylaxis is recommended, and scoring systems are helpful in early detection. In cancer patients with severe acute respiratory syndrome coronavirus 2, individual risk for thromboembolic events should be taken into account when considering interruption versus continuation of antitumoral therapy. However, further data and studies are required.

## Introduction

The world is experiencing a rapid spread of a novel virus, known as severe acute respiratory syndrome coronavirus 2 (SARS-CoV-2) [1]. By 1 November 2020, the World Health Organization reported 45,968,799 confirmed cases worldwide and 1,192,911 deaths due to coronavirus disease 2019 (COVID-19). Clinical symptoms partially overlap with other viral diseases: The most commonly reported symptoms are fever and cough as well as dyspnea, myalgia, confusion, diarrhea, nausea, and vomiting [2, 3]. The clinical course of the disease varies widely from asymptomatic to lethal [4]. Among others, male sex, older age, smoking, and presence of preexisting health conditions are described to adversely affect outcomes [5–9]. Cancer patients might be at increased risk of infection because of their higher morbidity as well as for being immunocompromised. They have repeatedly been reported to have a poorer clinical outcome [10, 11], especially in the setting of metastatic disease or recent surgery [12]. Regarding cancer in women, Mehta *et al*. reported a mortality rate of 14% for COVID-19 patients suffering from breast cancer and of 38% for patients suffering from pelvic gynecologic cancers [13].

As malignancies may affect all three arms of Virchow’s triad, patients with active cancerous disease are at elevated risk for venous thromboembolism (VTE) [14]. Presumably, 20–30% of all first thromboembolic events are cancer associated, making malignancies one of the best-established risk factors for VTE [15].

Recently, there has been growing evidence that the incidence of thromboembolic complications in critically ill COVID-19 patients is remarkably high [16–20]. Angiotensin-converting enzyme 2 (ACE2) is recognized as an important receptor used by SARS-CoV2 [21]. ACE2 protein is eminently expressed on arterial and venous endothelial cells. There is evidence of direct viral infection of endothelial cells [22] as well as indicators for microvascular injury syndrome mediated by activation of complement pathways [23]. Both can explain a procoagulant state in COVID-19 patients. Abnormal coagulation parameters, such as elevated D-dimer levels, are found to be associated with poor prognosis for COVID-19 patients [24–26]. Conversely, patients with a severe course of COVID-19 are more frequently diagnosed with disseminated intravascular coagulation [27].

The interrelationship between cancer-related hypercoagulopathy and SARS-CoV-2 in oncological patients therefore requires special attention. Here, we report the case of a patient suffering from high-grade serous ovarian cancer (HGSOC) who was admitted to our hospital with pulmonary embolism (PE) and COVID-19 during the SARS-CoV-2 pandemic in April 2020. To give an overview of what is known about the combination of a gynecologic malignancy, a VTE event, and COVID-19, we summarize available literature and elaborate on possible problems.

## Case report

A 71-year-old Caucasian woman, gravida 2/para 2, presented with pain in her lower left leg accompanied by progressive dyspnea in April 2020. She had already received dabigatran prescribed by her general practitioner for suspected deep vein thrombosis (DVT) one day prior to her admittance.

The patient was undergoing multimodal treatment at our clinic for HGSOC FIGO IIIC. Up to that point, she had received debulking surgery with macroscopic complete tumor resection in February 2020, including bilateral adnexectomy, hysterectomy, peritonectomy, infragastric omentectomy, pelvic and paraaortic lymphadenectomy, partial liver resection, and rectosigmoid resection. Secondary wound closure could be achieved after treatment of wound dehiscence in March 2020. First-line chemotherapy was initiated with carboplatin AUC 5 and paclitaxel 175 mg/m^2^ every 3 weeks. After completion of the first course, we switched to weekly application of carboplatin AUC 2 and paclitaxel 80 mg/m^2^ to improve tolerability. Three weekly courses of chemotherapy were administered without complications. Initiation of bevacizumab was postponed due to delayed wound healing. The patient reported a general feeling of weakness since surgery but was otherwise in a good state of health.

Her medical history included papillary thyroid cancer, successfully treated in 2008 by total thyroidectomy, and catheter ablation of the AV node because of atrioventricular nodal reentry tachycardia in 2015. No other relevant cardiovascular or pulmonal diseases were known. She had an uneventful gynecological and obstetrical history. *BRCA* testing was negative, and there was no family history of gynecologic malignancies. The patient did not smoke or drink alcohol regularly. She was taking l-thyroxin, pantoprazole, and vitamin D as regular medication.

At current presentation, she was routinely asked for symptoms and risk factors for COVID-19 and negated symptoms such as fever, cough, headaches, loss of taste or smell, or recent international travel. Her only contacts were close relatives as well as her general practitioner. Clinical examination showed normal clinical conditions as well as normal vital functions (blood pressure 123/75 mmHg, temperature 36.9 °C, heart rate 87 /minute, oxygen saturation 98 %) but a slightly elevated respiratory rate (22 /minute). Besides chronic anemia (hemoglobin 9.0 g/dl) and elevated infection parameters (C-reactive protein 8.1 mg/dl, ferritin 470 ng/ml), blood testing showed no relevant changes; especially, renal and hepatic function were normal. Sonography revealed a mild urinary stasis on the left side.

**Fig. 1:**
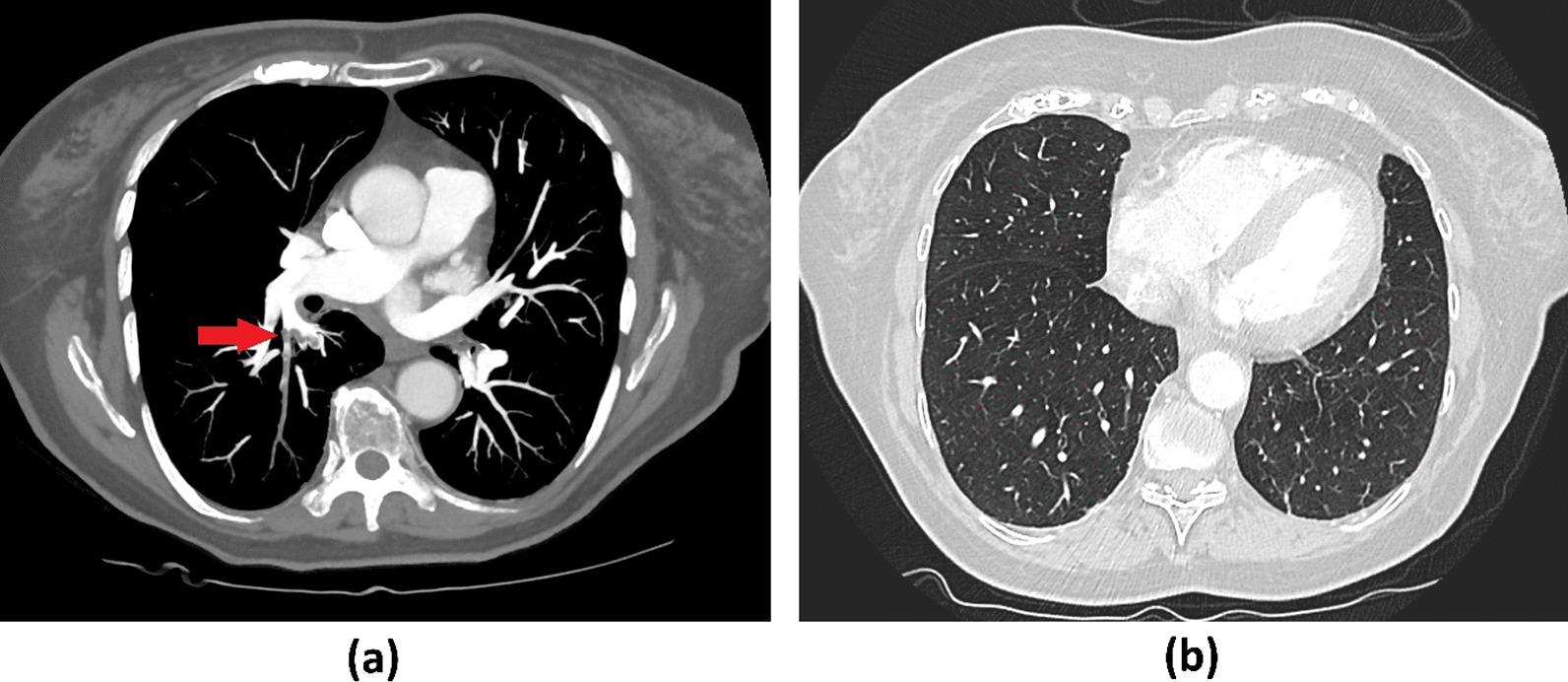
Computer tomography of the thorax: **a** arterial phase revealing a segmental PE in one central lung artery (red arrow) as well as in several subsegmental arteries, **b** lung window showed no abnormal findings, especially no changes typical for COVID-19

For further diagnosis, we referred the patient to the angiology department of our clinic, where DVT of the left lower leg as well as a thrombophlebitis of the right great saphenous vein and venous thrombosis of the right gastrocnemius muscle vein were confirmed via color Duplex sonography. We ordered chest computed tomography due to complaints of progressive dyspnea, revealing segmental PE in one central lung artery as well as in several subsegmental arteries (Fig. 1a). No infiltrates, especially no changes typical for COVID-19, were seen (Fig. 1b). We performed a nasopharyngeal swab as a routine screening test for SARS-CoV-2 upon hospitalization. Surprisingly, it revealed infection with SARS-CoV-2 via reverse-transcription polymerase chain reaction (PCR) testing on the following day. The woman was therefore isolated and transferred to the infectiology department of our clinic, where she was treated for ten more days. As the patient was in a stable cardiopulmonal condition with no signs of right heart overload in echocardiography, conservative treatment with low-molecular-weight heparins (LMWH) at a therapeutic dosage and compression stockings was continued. Respiratory parameters were sufficient with no need for oxygen therapy or further medication at any time. An incidental urinary tract infection with *Klebsiella pneumonia* was sufficiently treated with cefpodoxim 400 mg per day for 10 days.

A repeat PCR test before discharge was negative. The patient was quarantined at home for 14 more days. Anticoagulant treatment was continued with apixaban for 6 months. No further thromboembolic events occurred meanwhile. With a therapeutic gap of 5 weeks, we continued chemotherapy with the weekly regimen. For the next 2 courses, dose reduction to 75% was performed; full doses of carboplatin AUC 2 and paclitaxel 80 mg/m^2^ were resumed thereafter and well tolerated. A lymphocele of the left lower abdomen which had caused progressive urinary stasis was drained complication-free 5 months later. As bevacizumab was contraindicated because of prior VTE, oncologic maintenance therapy with niraparib 200 mg per day followed afterwards.

## Methods

**Fig. 2:**
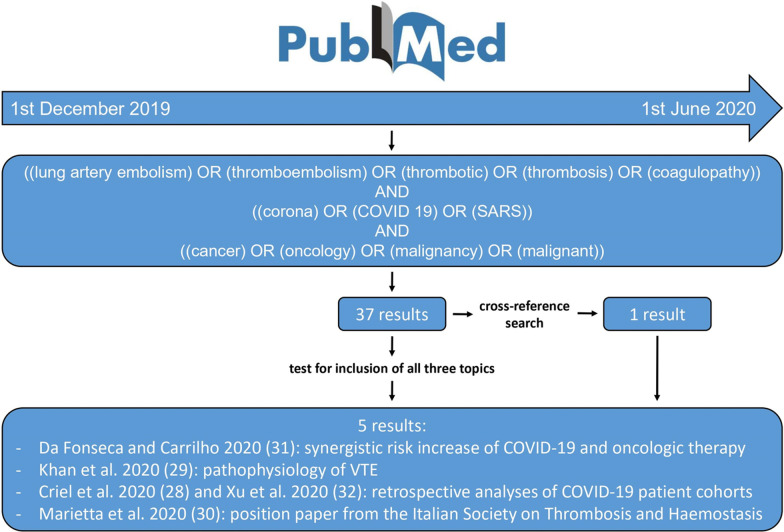
Flowchart of the systematic review of literature: According to PRISMA guidelines, we performed a comprehensive bibliographic search of PubMed limited to articles published from 1 December 2019 to 1 June 2020. Articles ahead of print were also included, and a cross-reference search of all relevant articles was carried out

For the systematic literature review according to PRISMA guidelines, we performed a comprehensive bibliographic search of PubMed limited to articles published from 1 December 2019 to 1 June 2020, using the following string term: [(lung artery embolism) OR (thromboembolism) OR (thrombotic) OR (thrombosis) OR (coagulopathy)) AND ((corona) OR (COVID 19) OR (SARS)] AND [(cancer) OR (oncology) OR (malignancy) OR (malignant)]. We did not limit our search to gynecologic malignancies due to the paucity of data. For the same reason, we also included articles ahead of print at the current time. The search generated 37 results, whose full texts were evaluated for relevance with respect to all three topics. Subsequently, we performed a cross-reference search of all relevant articles (Fig. 2).

## Results

The majority of articles were excluded as they did not focus on associating all three topics, or only briefly mentioned or cited one of the selected search terms without going into detail. Four research articles remained, including a retrospective study [28], a review on risk of thromboembolism in COVID-19 patients [29], a position paper on COVID-19 and hemostasis [30], as well as a letter to the editor referring to the risk of thrombotic events during systematic oncological treatment in COVID-19 patients [31]. The cross-reference search revealed one more retrospective study on risk assessment of VTE on COVID-19 patients meeting the inclusion criteria [32] (Fig. 2).

Xu *et al*. investigated the role of risk factors for VTE on clinical outcomes of 138 COVID-19 patients in a retrospective study. They found that critically ill patients are more likely to have malignancy as risk factor than non-critically ill patients (6.7% versus 2.4%), and the same could be obtained in patients with active cancer (6.7% versus 0.0%) [32].

Furthermore, Criel *et al*. reported 52 cases of COVID-19 patients receiving general inpatient care, two of whom had insidious VTE of the upper and lower limbs. One of these two patients suffered from active stage IV cancer of unreported origin. However, presence of PE was not investigated in this study. The incidence of thromboembolic events may therefore be underestimated [28].

Khan *et al*. focused on the diagnosis of VTE, discussing possible pathomechanisms in COVID-19 patients. The authors further outline the need for scoring systems to more reliably detect VTE, especially if risk factors such as malignancies preexist. They also give an overview about different challenges and management of VTE in COVID-19 patients and recommend generous use of thromboprophylaxis to improve patients’ outcome [29].

The Italian Society on Thrombosis and Haemostasis gives specific recommendations concerning the treatment of hemostasis imbalance in COVID-19 patients based on expert consensus. As patients with active cancer face an increased risk of VTE, thromboprophylaxis is suggested not only for the entire duration of their hospital stay but also in the time preceding and following it. The use of intermediate-dose LMWH should be evaluated based on individual risk factors [30].

The letter to the editor discusses an infection with SARS-CoV-2 in cancer patients treated with prothrombotic drugs such as tyrosine kinase inhibitors. As a possible synergistic risk increase for VTE should be avoided, timely interruption of treatment with prothrombotic drugs in patients with suspected SARS-CoV-2 infection must be considered [31].

## Discussion

We report herein a case demonstrating the relevance of elevated risk of VTE in cancer patients within the context of the ongoing SARS-CoV-2 pandemic. The systematic literature review illustrated that research into this topic is still in its infancy.

Several studies indicate that cancer patients suffering from COVID-19 face a less favorable prognosis, marked by increased requirement of intensive care treatment and mechanical ventilation as well as increased mortality [10, 11]. Breast cancer and malignancies of the female reproductive tract accounted for more than 38.6% of new cases of cancer among women and were responsible for over 29.1% of deaths resulting from cancer in 2018 [33]. Due to their incidence and their intrinsically high mortality rate, gynecologic malignancies are of particular interest in the context of SARS-CoV-2.

Leaving aside her underlying oncologic condition, despite infection with SARS-CoV-2, our patient was not in a critical health condition but rather presented without any typical symptoms of COVID-19 apart from mild dyspnea and tachypnea. Indeed, 17.9–20.8% of patients were described to be asymptomatic in recent studies [34, 35]. The authors suspect this to be due to a weaker immune response to SARS-CoV-2. However, it is unclear whether the putative increase of thrombotic complications associated with COVID-19 is less likely in asymptomatic or mild symptomatic patients as our case demonstrates a syncronous occurrence.

Interestingly, Miyashita *et al*. recently reported that cancer patients required invasive mechanical ventilation more frequently, but were less likely to die from COVID-19, albeit not to a statistically significant extent [36]. Some researchers attribute this to the impaired immune system of oncologic patients decreasing the risk of a hyperinflammatory syndrome [36, 37], which is discussed as a relevant cause of death in COVID-19 patients [38, 39].

Still, other authors reported elevated mortality rates for cancer patients [10, 12]. According to our findings, this may be explained by an increased risk for thromboembolism: We identified two studies reporting retrospective data, suggesting that VTE might worsen the outcome of oncologic patients or might even unfavorably influence their prognosis in case of SARS-CoV-2 infection [28, 32]. However, we found that the data available so far are insufficient and do not allow for a reliable interpretation of the role of hypercoagulability in this context.

Presumably, due to the rudimentary state of research, no specific data concerning gynecologic tumors are available yet. However, more information on this topic is urgently needed, as ovarian and uterine cancers are described to be associated with a comparatively high risk for VTE compared with other types of cancer [40]. In a study by Saadeh *et al*., the risk for VTE in patients with ovarian cancer was up to 9.7% [41]. Aside from a more advanced stage and a higher tumor grade, undergoing complex surgery and chemotherapy are relevant risk factors. Consequently, our patient already had a high risk of VTE. It is not possible to ascertain whether infection with SARS-CoV-2 played a decisive role, but considering the findings of the literature review, at least a synergistic mechanism is certainly conceivable. Consequently, it has to be assumed that administration of bevacizumab, a humanized anti-vascular endothelial growth factor (VEGF) monoclonal antibody with high prothrombotic potential [42, 43], would have further increased the risk of VTE and might have worsened the clinical outcome. A possible additional detrimental effect from oncologic drugs should be taken seriously when discussing COVID-19 in cancer patients. Potential benefits of interrupting oncological therapy in suspected cases as described by da Fonseca and Carrilho [31] need to be weighed against possible risks.

Using scoring systems for risk stratification as well as detection of VTE in COVID-19 patients in a timely manner as suggested by Khan *et al*. [29] seems to be a useful proposal in this and any other setting. The assumed elevated risk of cancer patients for VTEs has already attracted attention and led to specific recommendations concerning anticoagulant treatment in case of SARS-CoV-2 infection [30]. So far, these recommendations are based on expert consensus, and further data from clinical trials are needed to gain higher evidence. For instance, COVID-19 patients are reported to develop life-threatening thrombotic complications despite anticoagulant thromboprophylaxis [20]. On the other hand, there is promising data suggesting an improved outcome of hospitalized patients with COVID-19 receiving systemic anticoagulation at therapeutic doses [44]. Indeed, the potential benefits of anticoagulation need to be weighed against the risk of bleeding, which is increased in cancer patients, in particular after large surgical interventions, and would be increased further by anticoagulation [45].

Many authors have noted that the major risk for both cancer and VTE patients might be the inability to receive necessary medical services during this pandemic [46]. Consequently, the possibility of reduced quality of care might result in a deterioration of mid- and long-term results of cancer treatment [47].

## Conclusion

The reported case illustrates that cancer patients are at special risk regarding morbidity and mortality during the SARS-CoV-2 pandemic. Relevant factors in this context appear to be the combined prothrombotic potential of the viral disease, the oncologic disease itself, and the accompanying drug therapy. This applies to gynecologic tumors in particular, which account for a substantial part of incidence and mortality due to malignant disease in women. Therefore, they are of special interest in this context.

To carefully weigh opportunities and risks and to give reliable therapy recommendations for cancer patients during the SARS-CoV-2 pandemic, more data and studies are required. Already established international registries are an important step in this direction [48].

## Data Availability

Not applicable.

## Abbreviations

COVID-19: Coronavirus disease 2019
DVT: Deep vein thrombosis
HGSOC: High-grade serous ovarian cancer
LMWH: Low-molecular-weight heparins
PE: Pulmonary embolism
SARS-CoV-2: Severe acute respiratory syndrome coronavirus 2
VEGF: Vascular endothelial growth factor
VTE: Venous thromboembolism
